# Vagus Nerve Stimulation in Rodent Models: An Overview of Technical Considerations

**DOI:** 10.3389/fnins.2019.00911

**Published:** 2019-09-04

**Authors:** Crystal M. Noller, Yaakov A. Levine, Timur M. Urakov, Joshua P. Aronson, Mark S. Nash

**Affiliations:** ^1^The Miami Project to Cure Paralysis, Miller School of Medicine, University of Miami, Miami, FL, United States; ^2^Section of Neurosurgery, Department of Surgery, Dartmouth-Hitchcock Medical Center, Lebanon, NH, United States; ^3^Geisel School of Medicine, Dartmouth College, Hanover, NH, United States; ^4^SetPoint Medical Corporation, Valencia, CA, United States; ^5^Department of Neurological Surgery, Miller School of Medicine, University of Miami, Miami, FL, United States; ^6^Jackson Memorial Hospital, Miami, FL, United States; ^7^Department of Physical Medicine and Rehabilitation, Miller School of Medicine, University of Miami, Miami, FL, United States

**Keywords:** vagus nerve stimulation, vagus nerve, neuromodulation, nerve cuff electrode, electrical stimulation

## Abstract

Over the last several decades, vagus nerve stimulation (VNS) has evolved from a treatment for select neuropsychiatric disorders to one that holds promise in treating numerous inflammatory conditions. Growing interest has focused on the use of VNS for other indications, such as heart failure, rheumatoid arthritis, inflammatory bowel disease, ischemic stroke, and traumatic brain injury. As pre-clinical research often guides expansion into new clinical avenues, animal models of VNS have also increased in recent years. To advance this promising treatment, however, there are a number of experimental parameters that must be considered when planning a study, such as physiology of the vagus nerve, electrical stimulation parameters, electrode design, stimulation equipment, and microsurgical technique. In this review, we discuss these important considerations and how a combination of clinically relevant stimulation parameters can be used to achieve beneficial therapeutic results in pre-clinical studies of sub-acute to chronic VNS, and provide a practical guide for performing this work in rodent models. Finally, by integrating clinical and pre-clinical research, we present indeterminate issues as opportunities for future research.

## Introduction

Vagus nerve stimulation (VNS) is an FDA-approved treatment for select neurological and psychiatric conditions including epilepsy, treatment-resistant depression, and cluster headache (Heck et al., 2002; Ruffoli et al., 2011; Conway et al., 2016; Pisapia and Baltuch, 2016; Lainez and Guillamon, 2017; Kumar et al., 2019). There is also growing interest in using VNS to treat other conditions, such as heart failure, rheumatoid arthritis, inflammatory bowel disease, ischemic stroke, and traumatic brain injury (Zhang et al., 2009; Bonaz et al., 2013; Cai et al., 2014; Levine et al., 2014a,b, Levine et al., 2018a,b; Dawson et al., 2016; Guiraud et al., 2016; Koopman et al., 2016; Pruitt et al., 2016; Kanashiro et al., 2018), many of which are characterized by inflammation. Extensive pre-clinical evidence has demonstrated the utility of VNS in treating inflammatory conditions (Huston et al., 2006, 2007; Rosas-Ballina et al., 2008; Levine et al., 2014a,b; Olofsson et al., 2015), and a recent clinical study of rheumatoid arthritis (Koopman et al., 2016) further supports this treatment for widespread application. As VNS is applied to a broader range of conditions, it is important to recognize factors that influence study outcomes, such as stimulation settings, vagus nerve physiology, anatomical location of the target nerve branch, and electrode design. However, in many reports published thus far, these factors are often either not discussed, or are described as “customized” (i.e., as in the case of electrode design). A comprehensive discussion is therefore needed to inform the scientific design and reproducible execution of VNS studies.

The current review will provide a stepwise overview to inform the administration of VNS in rodent models, which often form the basis for higher-order study models. Key experimental conditions are discussed, including vagus nerve physiology, electrode design, stimulation equipment, microsurgical technique, and electrical stimulation parameters. Each step includes a detailed rationale to help inform modifications. Although a recent article outlined the surgical procedure for acute rodent VNS (Le Maitre et al., 2017), that method involved only a single stimulation and subsequent removal of the electrode. The current protocol will extend this work by outlining all of the steps necessary to conduct a full-scale sub-acute to chronic VNS study with an implanted cuff electrode. Here, we offer a practical guide to support pre-clinical VNS testing, anticipating the application of VNS for new clinical indications.

## Targeted Stimulation of the Vagus Nerve

### Vagus Nerve Physiology

The vagus nerve is the tenth and longest cranial nerve and the primary mediator of the parasympathetic branch of the autonomic nervous system (Tracey, 2002; Bonaz et al., 2013). It also regulates immune system homeostasis through an intrinsic “cholinergic anti-inflammatory pathway” (Tracey, 2002; Bonaz et al., 2013). The vagus is a mixed nerve, largely composed of afferent sensory (∼80%) and efferent motor (∼20%) nerve fibers (George et al., 2003, 2004; Groves and Brown, 2005), the composition differing depending on the anatomical location of the nerve (Prechtl and Powley, 1990). The vagus nerve contains three main fiber types: A-, B-, and C-fibers, which are distinguished by fiber diameter, myelination, and activation thresholds (Heck et al., 2002; Groves and Brown, 2005; Ruffoli et al., 2011). A-fibers are large and myelinated (5–20 μm in diameter) and are activated by the lowest amount of current (0.01–0.2 mA) (Schnitzlein et al., 1958; Groves and Brown, 2005; Vuckovic et al., 2008; Ruffoli et al., 2011). B-fibers are mid-sized and myelinated (∼1–3 μm in diameter) and are also activated by low currents (0.04–0.6 mA) (Schnitzlein et al., 1958; Groves and Brown, 2005; Vuckovic et al., 2008; Ruffoli et al., 2011). C-fibers, which constitute the majority of vagus nerve fibers (∼65–80% of afferent fibers), are small and unmyelinated (0.4–2 μm in diameter) and require the highest activation currents (greater than 2.0 mA) (Schnitzlein et al., 1958; Heck et al., 2002; Groves and Brown, 2005; Vuckovic et al., 2008; Ruffoli et al., 2011; Yoo et al., 2013). Although the distribution of the vagus nerve has been shown to be comparable in some species (Mackay and Andrews, 1983; Asala and Bower, 1986), the morphology of the nerve changes depending on the anatomical location (Agostoni et al., 1957; Hoffman and Schnitzlein, 1961; McAllen and Spyer, 1978; Mei et al., 1980; Jammes et al., 1982; Powley et al., 1983; Prechtl and Powley, 1990; Henry, 2002; Ruffoli et al., 2011; Clancy et al., 2013; Hammer et al., 2015, 2018; Verlinden et al., 2015; Bonaz et al., 2016; Yuan and Silberstein, 2016; Planitzer et al., 2017).

### Anatomical Considerations

Choosing an appropriate anatomical location to deliver stimulation is important when designing a VNS study. In most cases, VNS is delivered to the cervical vagus nerve (George et al., 2004; Howland, 2014) using an implanted electrode. Clinically, this anatomical location is the most common site for immune-modulation and treating epilepsy and depression (Lomarev et al., 2002; Nemeroff et al., 2006; Ben-Menachem et al., 2015; Koopman et al., 2016; Giordano et al., 2017). The left and right cervical branches differentially innervate the heart (O’Toole et al., 1986; Berthoud and Neuhuber, 2000; Ruffoli et al., 2011), where the right vagus nerve has more direct projections to the cardiac atria (Henry, 2002; Groves and Brown, 2005) and thus has a greater influence on heart rate. For this reason, left-sided VNS has been recommended to avoid adverse cardiovascular effects in humans (Henry, 2002; Groves and Brown, 2005; Van Leusden et al., 2015), even though equivalent anti-seizure effects are observed with either left, or right cervical VNS (Krahl et al., 2003; Navas et al., 2010). In rodent models, this lateral difference is not as clear and may differ depending on the stimulation parameters used (Stauss, 2017).

Anatomical differences in vagus innervation may be useful to help researchers adjust stimulation parameters to achieve specific, clinically relevant outcomes. Although most clinical applications target the left cervical vagus nerve, certain conditions may benefit from targeting different anatomical branches. For example, VNS applied to the right cervical vagus nerve is currently being explored to treat heart failure, where direct cardiac effects are desired (Li et al., 2004; Howland, 2014). Morphological differences in the right and left cervical vagus nerve branches (Verlinden et al., 2015) may also explain different clinical effects. Specifically, it was recently reported that both cervical nerve branches contain tyrosine hydroxylase- and dopamine β-hydroxylase-positive nerve fibers, but that the right cervical branch has a larger surface area and double the number of tyrosine hydroxylase-positive nerve fibers (Verlinden et al., 2015). These findings may inform the use of VNS for select cholinergic or adrenergic effects (Onkka et al., 2013; Seki et al., 2014; Verlinden et al., 2015).

Anatomical differences between the cervical vagus nerves may be less important in the treatment of other conditions, likely due to the abundant crossover of fibers between branches of the vagus nerve (Berthoud and Neuhuber, 2000). For example, in a recent study, no significant differences in inflammatory cytokines were found in animals receiving unilateral VNS to the left cervical vagus nerve compared to those receiving bilateral stimulation (VNS applied to the right and left nerve branches) (Olofsson et al., 2015). Laterality concerns may also be less pertinent for the emergent interest in transcutaneous VNS at the auricular or cervical branch (Howland, 2014; Ben-Menachem et al., 2015) and subdiaphragmatic VNS (Greenway and Zheng, 2007; de Lartigue, 2016). It remains to be determined whether the same stimulation parameters can be used for different vagus nerve branches or implanted vs. non-implanted modalities. Further research is needed to elucidate stimulation parameters for each clinical indication and comparative efficacy for implanted vs. non-implanted approaches. Here, the current review will focus on the most common clinical and pre-clinical stimulation site, the left cervical vagus nerve (Howland, 2014), using an implanted electrode. As rodents are commonly used in physiological studies with clinical relevance and form the basis for higher-order models, this overview will discuss specifications pertinent to mouse and rat models in a clinically relevant context, starting with electrode design and implantation, and concluding with stimulation parameters.

## Electrode Design

The electrode design is an important factor to consider when planning a VNS study. In the most common clinical deployment using a can-and-lead system, the cervical vagus nerve is encircled with bipolar helical electrodes, and a pulse generator is implanted in the chest wall (Bonaz et al., 2013; Giordano et al., 2017). The electrode configuration consists of two spiral electrodes placed around the vagus nerve: the cathode is placed cranial and the anode caudal. A third helical tethering anchor is also placed around the nerve, directly caudal to the anode to provide strain relief (Pisapia and Baltuch, 2016; Giordano et al., 2017).

**FIGURE 1:**
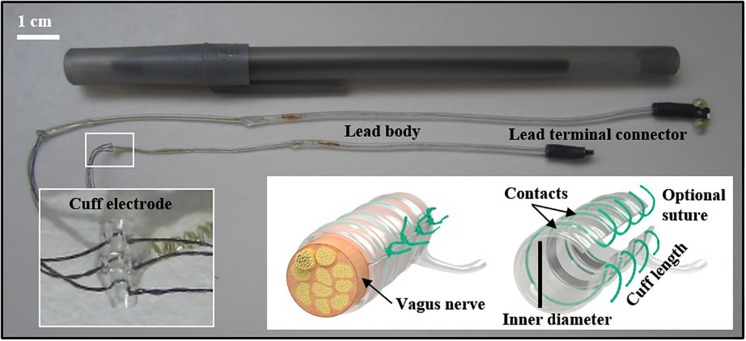
Different sized (0.3 and 0.5 mm) cuff electrodes with connectors. Embedded sutures on the electrode can facilitate the surgical implant (inset). Other important electrode elements described in the text are identified. Cuff image reproduced with permission from MicroProbes for Life Science (Gaithersburg, MD).

**FIGURE 2:**
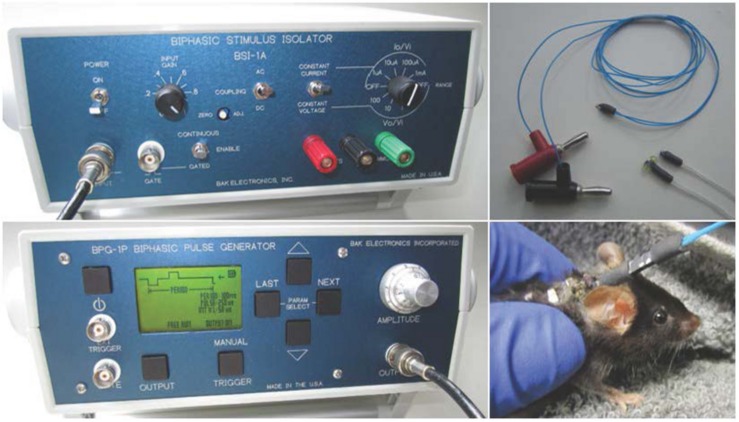
**Left panel** (top to bottom): Biphasic current-controlled stimulus isolator and biphasic pulse generator (BSI-1A, BPG-1P, respectively; Bak Electronics Incorporated, Umatilla, FL, United States). **Right panel** (top to bottom): Omnetics mating plug for the externalized connector and awake animal receiving stimulation. All procedures described and animal photography was performed with approval by the Institutional Animal Care and Use Committee (IACUC) at the University of Miami.

Electrodes used in rodents may differ, depending on the research objectives and study length (ranging from acute to chronic stimulation). The electrode configuration can include many designs (e.g., cuff or hook, and the inclusion of recording electrodes). A recent VNS methods paper described a needle electrode that was placed under the left cervical vagus nerve during a single stimulation (Le Maitre et al., 2017). For research involving multiple stimulation treatments, we implanted a bipolar cuff electrode with embedded sutures around the nerve (see Figure 1). Cuff electrodes are used in acute and chronic implantation to prevent current leakage into the surrounding tissue. We applied a strain-relieving suture close to the deployment site (detailed below), and the attached lead and pin connector was then tunneled under the skin and externalized at the base of the neck. Figure 2 depicts the externalized connector and an awake animal receiving stimulation. Stimulation was delivered with a commercially available external pulse generator and current-controlled stimulus isolator (see Figure 2), described below.

For control conditions (unstimulated), a sham electrode can be made by implanting a silicon tube that is the same size as the electrode. This relatively inexpensive inactive design is helpful for feasibility testing, as it compensates for the mechanical stimulation that occurs when the nerve is manipulated (Huston et al., 2007). However, other variables (e.g., the weight of the electrode and tension from the connecting wire-embedded lead) may influence study outcomes, and it is best to have a control condition that consists of an identical implanted electrode that does not receive electricity or a cuff and lead constructed without wires.

In animal experimentation, placement of the cathode and anode are typically not reported. Animal research often includes study factors not relevant to clinical practice, such as stimulation of the distal nerve trunk of a vagotomized animal (Borovikova et al., 2000; de Jonge et al., 2005). In a recent rodent study, no significant differences in inflammatory cytokines were found with either rostral or caudal placement of the cathodic lead (Olofsson et al., 2015), likely because action potentials are generated in both directions when an axon is depolarized. Additional research is needed to understand how cathodic placement and pulse parameters can be modified for specific treatment indications, a topic that is currently being explored (Ardell et al., 2017; Patel et al., 2017; Stauss, 2017).

### Electrode Specifications for Mice and Rats

Although many research groups construct their own electrodes, they can also be purchased and customized through commercial vendors. Nerve cuff electrodes can be made from several conductive materials, including platinum, platinum-iridium, and stainless steel. These electrodes are typically designed with monopolar, bipolar, or tripolar configurations, referring to the number of independent electrical contacts within the cuff. The tradeoff is generally increased control over current flow with a greater number of contacts, but also greater cost and cuff length. Monopolar electrodes are cheaper but require a return or ground, and current paths are less controllable. Bipolar electrodes are more expensive but allow better control over current flow than monopolar, as most of the current will flow directly between the two adjacent contacts. Bipolar cuff electrodes may be more practical and are widely used in pre-clinical research when the cost is a determining factor, especially in pilot studies. Tripolar electrodes may be connected in a pseudo-tripolar configuration (the two external electrodes linked together to form a common anode), which prevents current from leaking out of the cuff.

The size of the target nerve will determine the size of the electrode, as the inner diameter of the cuff should be approximately 1.4 times the outer diameter of the nerve. Use of a cuff that is too small could damage the nerve, and one that is too large could lead to insufficient surface contact or excessive current leakage (Agnew and McCreery, 1990; Yoshida and Riso, 2004). We used nerve cuffs with an inner diameter of 0.3 mm for both mice and small rats and 0.5 mm for larger rats. These sizes were informed by our experience and by published reports (Olofsson et al., 2015).

Besides inner diameter, other modifications include the number of contacts, the distance between contacts, and distance from the contacts to the end of the cuff. The number of contacts is determined by the stimulation paradigm desired. The distance between contacts (“inter-electrode spacing”) we used was 0.5 mm for mice and small rats and 1 mm for larger rats. The distance from the last contact to the end of the cuff is typically three times the distance between contacts to prevent significant current leakage outside the cuff. This distance can be much smaller in pseudo-tripolar electrode configuration. Other custom modifications can be made to suit the research experiment, for example, using an angled cuff design, a multichannel omnetics connector on the externalized cuff lead, embedded suture material on the cuff for convenient closure, suture rings exiting from the base of the connector, and protective silicone tubing around the wiring. A deep review of electrode design is beyond the scope of the current article, but has been reviewed extensively elsewhere (Loeb and Peck, 1996; Merrill et al., 2005; Foldes et al., 2011; Dweiri et al., 2016; Caravaca et al., 2017).

Finally, stimulation conditions in acute vs. chronic studies may differ, as it has been shown that connective tissue forms around the cuff within the first 2 months of implantation (Agnew and McCreery, 1990; Helmers et al., 2012). While this scar tissue will bind the nerve and cuff together and prevent movement abrasion, it can also increase the impedance, causing an increase in the amount of stimulation voltage required to excite the nerve (Agnew and McCreery, 1990; Helmers et al., 2012). Furthermore, it was recently shown that chronic cuffing of the vagus nerve changes the integrity of the nerve fibers, likely due to the inflammatory response to the foreign material (Somann et al., 2018). Although the inflammatory response should resolve upon the formation of fibrous tissue, in some instances, it can negatively affect the integrity of the nerve, including demyelination (Tyler and Durand, 2003; Thil et al., 2007; Somann et al., 2018). It is currently unknown whether this process affects afferent and efferent vagus nerve fibers equally, but it is a potential source of study variability that should be considered when planning a chronic VNS study (Somann et al., 2018). Recent advancements in cuff design show promise in addressing some of these concerns (Caravaca et al., 2017).

## Stimulation Equipment

**FIGURE 3:**
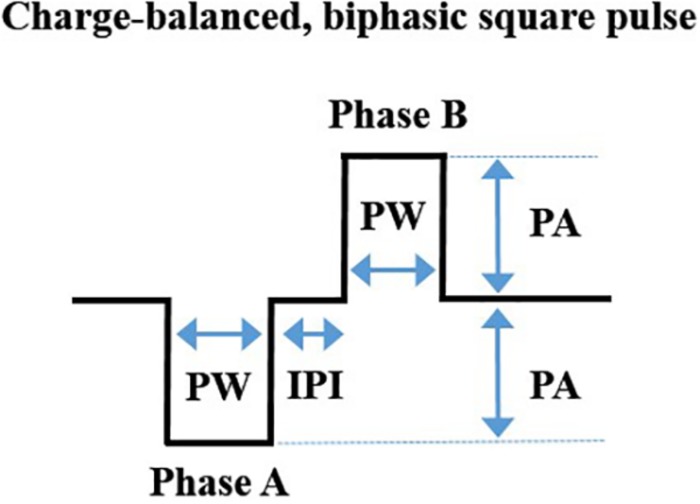
A depiction of the waveform of one charge-balanced, biphasic, cathodic-leading square pulse cycle, showing pulse width (PW), inter-phase interval (IPI), and pulse amplitude (PA). These components have been explained in detail elsewhere (Merrill et al., 2005). Image adapted with permission (Levine et al., 2014b; Olofsson et al., 2015) under the Creative Commons Attribution License.

Commercially available equipment can be used to deliver a charge-balanced, biphasic square-wave pulse to a bipolar cuff electrode (Levine et al., 2014b; Olofsson et al., 2015). For investigators wanting readily available equipment, we have outlined the specifications related to one vendor, Bak Electronics Incorporated (Umatilla, FL, United States), but many of these specifications are also relevant to other vendors. For our studies, we delivered biphasic (cathodic-leading) stimulation (Levine et al., 2014b; Olofsson et al., 2015) using an external biphasic pulse generator and current-controlled biphasic stimulus isolator (BPG-1P and BSI-1A, respectively). Gently restrained rodents (without anesthesia) were connected to the equipment with a mating plug for stimulation delivery (De Herdt et al., 2009; Zhang et al., 2009). See Figure 2 for equipment configuration and stimulation. Stimulation can be delivered for a specific period (e.g., 60 s) using the manual trigger or it can be digitally triggered or gated to a particular stimulus with a laptop configuration. The waveform design of one charge-balanced, biphasic, cathodic-leading square pulse cycle is represented in Figure 3.

The delivery specifications of each VNS “treatment” will depend on the physiological outcome measure to be studied. For example, in the context of anti-inflammatory effects, it has been shown that VNS delivered 24–48 h prior to endotoxin exposure resulted in a significant reduction in tumor necrosis factor (TNF) cytokine upon exposure (Huston et al., 2007). This period of therapeutic effects suggests that for certain indications (e.g., conditions characterized by an exaggerated inflammatory response to stimuli), VNS may be administered prophylactically or at specific intervals. As VNS treatment will likely be tailored to clinical symptoms, additional work is needed to determine the temporal response of VNS for other indications. Finally, it is important to include a measurable variable to determine stimulation effectiveness and rule out potential sources of study variability, such as incorrect placement or faulty electrodes, or mechanical damage to the nerve. One option is to record evoked potentials from the vagus nerve after VNS, for example, by stimulating the cervical branch and recording from the subdiaphragmatic branch (Olofsson et al., 2015). This approach can be taken just prior to euthanasia, or immediately after implantation; however, recording from the vagus nerve is technically challenging to perform in rodents (Silverman et al., 2018). To circumvent some of these challenges, investigators can record in a smaller cohort of animals, use electrodes that perform both stimulation and recording, or obtain electromyography (EMG) recordings of the laryngeal muscles that are innervated by the vagus. Another common option is to use heart rate to verify effective cuff placement, where stimulation is increased until a change in heart rate is observed (Levine et al., 2019). At a minimum, we recommend to visually inspect the nerve at euthanasia to ensure that it is still within the cuff and, when possible, to perform histology on the excised nerve to verify axon integrity.

## Microsurgical Technique: Electrode Placement

The stepwise surgical procedure detailed below involves the placement of an electrode on the LEFT cervical vagus nerve. The lateral configuration will change if implanting on the RIGHT nerve branch. All survival surgeries should be performed with aseptic technique while the animal is anesthetized, as recommended by the local Institutional Animal Care and Use Committee. Standard post-operative care should also be administered, including hydration and analgesia. With the animal lying prone, make a small opening (∼2 mm) in the skin at the craniovertebral junction, then place the animal in supine position. Palpate the sternal notch. Using scissors, carefully make a vertical incision at the neck, 3 mm caudal to the sternal notch. Dissect the subcutaneous space and extend the midline opening toward the jaw. Identify the inferior border of the thyroid tissue (“V” shaped distinct white line at the cervico-sternal junction). Blunt dissect the thyroid tissue along this line and reflect rostrally. Using a curved, narrow hemostat, create a subcutaneous tunnel toward the opening made at the craniovertebral junction. Bring the electrode through the tunnel and secure to the side of the incision site with tape or weighted instruments. Using blunt-tipped surgical hooks or modified needle syringes as hooks, reflect the thyroid superiorly and the left sternocleidomastoid inferiorly to expose the carotid fossa. No cutting of subcutaneous tissue should be done at any time.

**FIGURE 4:**
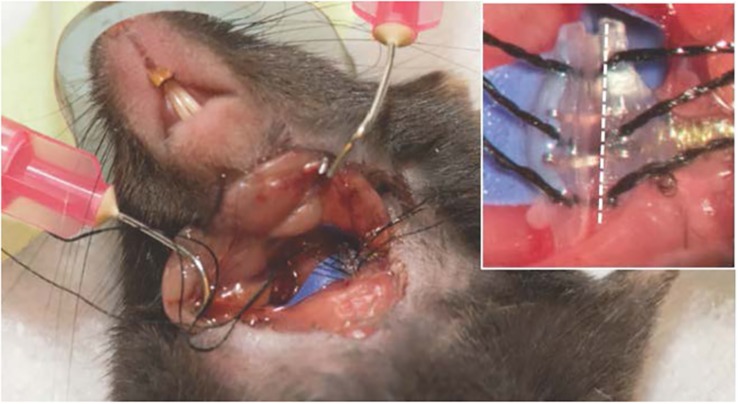
Surgical preparation of the electrode implant. Close up (inset), where the white dotted line lies parallel to the nerve lying inside the cuff. All procedures described and animal photography was performed with approval by the Institutional Animal Care and Use Committee (IACUC) at the University of Miami.

Isolate the left carotid sheath from the connective tissue using pickup forceps and gentle opening-closing movements parallel to the vessels. Identify the nerve (white in appearance) located directly behind or adjacent to the carotid artery. Note, gentle dissection can be accomplished with small, blunt-tipped forceps, minimizing the risk of vessel puncture; however, sharp forceps can also be carefully used to separate the nerve from the carotid artery. Gently dissect the nerve circumferentially taking care not to damage the vessels or tug on the nerve, and place background material under the nerve (a small piece of sterile glove will serve this purpose). Feed the suture strings from the same side of the electrode cuff under the nerve. Advance the electrode cuff under the nerve. Open the cuff by pulling on the opposing sutures, allow the nerve to slide into the open cuff, and secure the cuff by tying the suture strings (see Figure 4). If the visualized nerve is very thin, it is also possible to encircle the entire carotid sheath in mice without isolating the nerve (Olofsson et al., 2015). Attach the lead body to the subcutaneous tissue with a non-absorbable suture (e.g., 4–0 Webcryl) placed approximately 5 mm away from the cuff. This suture will secure the electrode and provide strain relief. With the animal lying prone, feed the excess encased wires evenly into the subcutaneous space (if necessary, create a pocket with forceps by gentle opening-closing movements). Secure the connector to the skin with non-absorbable suture (using embedded suture rings or suture carefully placed through the rubber encasement) and close the skin opening. Before closing the neck incision, place the animal back in a supine position to inspect the electrode and ensure the cuff is encircling the nerve without twisting or pulling. Close the incision on the neck. During surgery, care should be taken to avoid excessive manipulation of the nerve to prevent axonal damage, and, as has been reported, mechanical activation of neuroimmune reflexes (Huston et al., 2007).

## Electrical Stimulation Parameters

### Current FDA-Approved Clinical VNS Guidelines

Many of the lessons learned in the application of VNS to treat epilepsy are generalizable to other VNS applications, although stimulation should be optimized to the specific treatment condition. Several important parameters can be adjusted when delivering stimulation to the vagus nerve: output current, pulse width, pulse frequency, and duty cycle (i.e., “ON” and “OFF” time) (Heck et al., 2002; Groves and Brown, 2005; Wheless et al., 2018). Collectively, these parameters determine the total amount of electrical energy delivered to the vagus nerve during treatment (Heck et al., 2002). Current guidelines in epilepsy include output currents between 0.25 and 3.5 mA and pulse frequencies ranging between 20 and 30 Hz (Heck et al., 2002; Groves and Brown, 2005; Ruffoli et al., 2011). Continuously applied, high-frequency (50–100 Hz) current leads to irreversible axonal injury, which can be avoided by reducing the frequency to 20 Hz (Agnew and McCreery, 1990). These findings and others showing optimal pulse frequencies for seizure reduction ranging from 10 to 30 Hz (Woodbury and Woodbury, 1990; Zabara, 1992) led to current FDA-approved guidelines (Groves and Brown, 2005). These settings also correspond with stimulation parameters reported in clinical trials of depression (Rush et al., 2000, 2005a,b) and a recent clinical study of rheumatoid arthritis (Koopman et al., 2016). Once a target fiber type is identified, stimulation can be adjusted within approved limits.

The output current is the stimulation parameter typically adjusted first (Heck et al., 2002). A tolerable range of current can be used to target specific nerve fibers and achieve clinical efficacy. Initially, VNS treatment for clinical epilepsy was thought to activate C-fibers, an approach that coincided with observations of progressive anti-epileptic effects with increasing current (Heck et al., 2002). This approach was not without consequence, as elevated currents are less tolerable and increase adverse effects, such as bradycardia, dyspnea, and throat tightness (Heck et al., 2002). The need to increase output current to achieve clinical efficacy (DeGiorgio et al., 2000, 2001) has been questioned and may have been influenced by the high currents used for non-responders (Heck et al., 2002). It has since been shown that C-fiber activation is not required for anticonvulsant effects (Krahl et al., 2001; Henry, 2002; Ruffoli et al., 2011) and that currents above 2 mA may be unnecessary for most patients (Heck et al., 2002).

The second parameter to consider is the pulse width, the duration of a single stimulation pulse (Labiner and Ahern, 2007), which can be adjusted to avoid the neural damage associated with continuous stimulation (Agnew and McCreery, 1990). Rodent seizure models report optimal pulse widths as high as 1,000 μs; however, the pulse width is inversely related to tolerance in people (Heck et al., 2002). Fortunately, this parameter can be adjusted to improve tolerance without loss of efficacy, for example, by shortening the pulse width from 500 μs to 250 μs (Heck et al., 2002). Pulse width is inversely related to the current required to stimulate a nerve (Heck et al., 2002; Labiner and Ahern, 2007; Levine et al., 2019), and together these two parameters determine the total charge per pulse (Labiner and Ahern, 2007; Levine et al., 2019). Although shorter and longer pulse durations may be preferred in different clinical applications (Agnew and McCreery, 1990), this is an area of active investigation (Mu et al., 2004; Kong et al., 2012; Aaronson et al., 2013; Loerwald et al., 2018). Thus, adjusting pulse width in concert with output current can meet stimulation requirements and reduce the risks of excessive stimulation current, while achieving a balance of clinical efficacy and tolerability.

A third parameter, pulse frequency (number of pulses per second), is less often varied in studies compared to other stimulation settings. Pulse frequencies that are generally used in rodent seizure models are similar to those used clinically, ranging from 10 to 30 Hz (Heck et al., 2002). For treatment indications besides epilepsy, the utility of specific pulse frequencies remains under investigation. In clinical practice, it may be helpful to use the natural firing rates of specific fiber types to guide pulse frequency (Bonaz et al., 2013). For example, it has been reported that the physiological firing frequency of A- and C-fibers is above and below 10 Hz, respectively (Binks et al., 2001). It is currently unclear whether pulse frequency can be used to preferentially stimulate afferent vs. efferent fibers without the use of chemical or electrical nerve block (Osharina et al., 2006; Bonaz et al., 2013; Olofsson et al., 2015; Patel et al., 2017; Patel and Butera, 2018), as generally, when an axon is depolarized beneath a cathodic electrode, action potentials travel in both directions.

To date, pulse frequencies that are responsible for specific clinical effects remain to be determined; however, as more studies are performed, we will better understand whether a combination of stimulation settings will be useful in targeting specific nerve fibers within efferent and afferent pathways [e.g., motor A-type or sensory group I, II, and III fibers (Yoo et al., 2013)]. The use of different stimulation paradigms to selectively target different fiber types remains an area of active investigation (Vuckovic et al., 2004, 2008; Howland, 2014; Peclin and Rozman, 2014; Guiraud et al., 2016; Patel et al., 2017; Nuntaphum et al., 2018; Patel and Butera, 2018).

A final parameter that affects stimulation is the duty cycle (ON/OFF cycle) (Heck et al., 2002; Labiner and Ahern, 2007). The standard duty cycle to treat persons with epilepsy is 30 s ON/5 min OFF (Heck et al., 2002). Although shorter OFF times can improve clinical efficacy, rapid cycling (i.e., very short duty cycles) is much more energy intensive and may not be necessary for effective treatment (Heck et al., 2002), at least for anti-seizure use. A more in-depth discussion on considerations regarding electrical stimulation can be found in previous reviews (Merrill et al., 2005; Mortimer and Bhadra, 2009; Cogan et al., 2018; Grill, 2018). As an example using our equipment setup (Figure 2), a stimulation “treatment” consisting of symmetrical biphasic square pulses with current intensity of 500 μA, 250 μs pulse width, and 50 μs inter-phase interval at 10 Hz for 60 s (Olofsson et al., 2015) can be delivered with the following settings. *Stimulus isolator*: 100 μA of constant current, AC coupling, continuous stimulation, and an input gain of 1. *Pulse generator*: 5.0 amplitude (5 × 100 μA × gain of 1), 250 μs pulse width, 100 ms period. It should be noted that the pulse period is the inverse of the desired frequency (i.e., 100 ms = 1/10 Hz × 1000 ms/s). To summarize, the stimulation “treatment” using the parameters from our previous example will consist of a 550 μs pulse (250 μs for each phase of the pulse, plus 50 μs inter-phase interval), and a 99,450 μs inter-pulse interval (total of 100,000 μs period).

### Adverse Effects Associated With VNS

Commonly reported VNS adverse effects include cough, throat tightening or discomfort, shortness of breath, voice alterations, and cardiovascular symptoms, such as bradycardia (Ben-Menachem, 2001; Heck et al., 2002; Ben-Menachem et al., 2015; Jacobs et al., 2015). These transient effects are limited to when the device is actively stimulating and are proportional to increases in output current, pulse width, frequency, and duty cycle (Heck et al., 2002; Jacobs et al., 2015; Olofsson et al., 2015). Pulmonary effects are mainly associated with C-fiber activation (Banzett et al., 1999; Heck et al., 2002; Henry, 2002), which were previously associated with cardiovascular effects. However, it has been shown that cardioinhibitory effects can be attributed to activation of B-fibers (Jones et al., 1995; Banzett et al., 1999; Yoo et al., 2016; McAllen et al., 2018; Qing et al., 2018). Importantly, no evidence of “clinically relevant” bradycardia has been reported for stimulation within FDA-approved guidelines (Heck et al., 2002). It is also notable that anti-inflammatory and cardioinhibitory effects are separable (Huston et al., 2007), further indicating that stimulation parameters can be tailored for precise, clinically relevant outcomes.

### Tailoring VNS for Specific Conditions

Early anti-epileptic work targeted C-fibers under the assumption that these abundant afferent fibers mediate the clinical effects of VNS (George et al., 2003, 2004; Groves and Brown, 2005; Yoo et al., 2013). As such, early VNS treatment utilized the high output currents needed to stimulate C-fibers (Heck et al., 2002). As A-, B-, and C-fibers are successively recruited with increased electrical current (Woodbury and Woodbury, 1990; Yoo et al., 2013), subsequent research demonstrated anticonvulsant effects without specifically targeting C-fibers, thus allowing for smaller amounts of current (Krahl et al., 2001; Henry, 2002; Ruffoli et al., 2011). The activation thresholds of A- and B-fibers overlap, but both require substantially less current than C-fibers (Groves and Brown, 2005; Castoro et al., 2011), which, when activated, are associated with most of the reported adverse effects (Heck et al., 2002; Henry, 2002).

As the field has progressed, this iterative process of associating specific fiber types with therapeutic effects has occurred in the use of VNS for other indications, where anti-inflammatory effects have been attributed to A-fibers (Huston et al., 2007) and B-fibers (Olofsson et al., 2015). Importantly, recent research shows that minimal stimulation can achieve beneficial therapeutic outcomes. A seminal pre-clinical study demonstrated that a minimal amount of current (0.5 mA) activated the inflammatory reflex in both mice and rats (Olofsson et al., 2015), effects of which were observed up to 2.5 mA. These output currents remain below FDA-approved levels (Heck et al., 2002) and are similar to currents used clinically for rheumatoid arthritis (Koopman et al., 2016). Thus, minimized stimulation may provide therapeutic benefits while avoiding the adverse effects associated with higher output currents (Heck et al., 2002).

## Conclusion

Decades of research has brought extensive progress to the field of neuromodulation, and specifically to the clinical use of techniques such as VNS. Although VNS is a promising neuromodulation tool, it has been challenging to incorporate study findings from clinical and pre-clinical research. Pre-clinical VNS work often involves mechanistic study aspects not employed in clinical settings, such as the use of lidocaine to block efferent or afferent signaling or electrical stimulation of nerve stumps after vagotomy (Borovikova et al., 2000; de Jonge et al., 2005; Niederbichler et al., 2009; Olofsson et al., 2015). In clinical VNS application, decisions regarding stimulation parameters may not be explicitly defined, such as the number of treatment sessions; similarly, a clear rationale for pulse-design modifications are often not addressed. In addition to pulse frequencies, several other key stimulation parameters can influence study outcomes and reproducibility; however, selection of specific treatment parameters are often not detailed or are simply reported as “customized.” Furthermore, as activation thresholds may differ depending on conditions of stimulation, it is critical that study conditions are outlined in ongoing research efforts.

These issues highlight the challenge of translating rodent work to clinical application. The inflammatory reflex appears to be conserved across species; where anti-inflammatory effects are observed with similar stimulation parameters in rodents with endotoxemia (Olofsson et al., 2015), rodents with collagen-induced arthritis (Levine et al., 2014b), and persons with rheumatoid arthritis (Koopman et al., 2016). However, it is unknown whether specific fiber types that mediate the reflex are similarly conserved. Additionally, as disease-related factors may influence VNS pathways, stimulation may be most effective if delivered at specific time points of disease progression. These and other questions remain to be determined and highlight the importance of translating pre-clinical findings to heterogeneous clinical populations.

The current review aims to advance VNS research by providing a comprehensive discussion on performing pre-clinical VNS studies in rodent models. We have provided a microsurgical technique, discussed stimulation equipment, and provided a rationale for choosing electrode design and electrical stimulation settings. We outlined how a combination of clinically relevant stimulation parameters can be adjusted to achieve selected therapeutic effects. Indeterminate issues are discussed and presented as avenues for future research.

## Author Contributions

CN, YL, TU, JA, and MN conceived, structured, and participated in writing and revising the review.

## Conflict of Interest Statement

YL is an employee of SetPoint Medical Corporation, a company that is developing bioelectronic devices to target the vagus nerve in humans. The remaining authors declare that the research was conducted in the absence of any commercial or financial relationships that could be construed as a potential conflict of interest.
